# Risk Factors Associated with Early and Late Postoperative Complications in Neonatal Patients with Esophageal Atresia

**DOI:** 10.3390/children12081075

**Published:** 2025-08-15

**Authors:** Misela Raus, Luka Zekovic, Sanja Sindjic-Antunovic, Predrag Rodic, Biljana Medjo, Ivana Bosiocic, Aleksandar Dimitrijevic, Dejan Nikolic

**Affiliations:** 1 Department of Neonatology, University Children’s Hospital, 11000 Belgrade, Serbia; luka.zekovic@udk.bg.ac.rs; 2 Faculty of Medicine, University of Belgrade, 11000 Belgrade, Serbia; sanja.sindjic@udk.bg.ac.rs (S.S.-A.); predrag.rodic@udk.bg.ac.rs (P.R.); biljana.medjo@udk.bg.ac.rs (B.M.); aleksandar.dimitrijevic@udk.bg.ac.rs (A.D.); dejan.nikolic@udk.bg.ac.rs (D.N.); 3 Department of Neonatal Surgery, University Children’s Hospital, 11000 Belgrade, Serbia; 4 Department of Hematology, University Children’s Hospital, 11000 Belgrade, Serbia; 5 Department of Pediatric Intensive Care Unit, University Children’s Hospital, 11000 Belgrade, Serbia; 6 Department of Neurology, University Children’s Hospital, 11000 Belgrade, Serbia; ivana.bosiocic@udk.bg.ac.rs; 7 Department of Physical Medicine and Rehabilitation, University Children’s Hospital, 11000 Belgrade, Serbia

**Keywords:** early postoperative complications, late postoperative complication, esophageal atresia, neonates, risk factors

## Abstract

**Highlights:**

**What are the main findings?**

- A risk factor for early postoperative complications in neonates that have undergone surgical intervention for esophageal atresia is complications during delivery.
- The risk factors for late postoperative complications in neonates that have undergone surgical intervention for esophageal atresia are preoperative mechanical ventilation, postoperative sepsis, and belonging to relatively high- and high-risk groups according to the Spitz classification.

**What are the implications of the main findings?**

- Neonates with esophageal atresia and complications during delivery should be monitored during the perioperative period to promptly recognize or prevent the onset of early postoperative complications.
- In the postsurgical period, neonates with esophageal atresia who have been on preoperative mechanical ventilation or diagnosed with postoperative sepsis or who belong to relatively high- and high-risk groups according to the Spitz classification should be continuously monitored to promptly prevent or recognize late postoperative complications.

**Abstract:**

**Background and Aim:** Atresia is the most common congenital anomaly of the esophagus, with an increased risk of complications after surgical correction. The aim of our study was to evaluate the risk factors associated with early and late postoperative complications in neonatal patients with esophageal atresia. **Methods:** The study sample comprised 109 neonatal patients aged between 0 and 27 days of life who were prenatally diagnosed with esophageal atresia or diagnosed at birth. For the purpose of this study, neonatal and perinatal factors and factors associated with the mother’s medical condition were analyzed. Complications after surgical intervention were classified as early and late. **Results:** Patients with early postoperative complications experienced significantly more frequent complications during delivery (*p* = 0.002), asphyxia (*p* = 0.038), and postoperative sepsis (*p* = 0.045) and were more likely to have received medicamentous therapy (*p* = 0.035). Patients with late postoperative complications had significantly more frequent complications during delivery (*p* = 0.025), respiratory distress (*p* = 0.043), and postoperative sepsis (*p* = 0.010), were more likely to have received preoperative mechanical ventilation (*p* = 0.014), and showed a significantly different frequency distribution among the different classes of the Spitz classification (*p* = 0.008). A risk factor for early postoperative complications in patients with atresia in the upper part was complications during delivery (OR-3.09; *p* = 0.007). The risk factors for late postoperative complications for patients with upper atresia were preoperative mechanical ventilation (OR: 2.77; *p* = 0.041), postoperative sepsis (OR: 2.60; *p* = 0.028), and belonging to relatively high- and high-risk groups according to the Spitz classification (OR: 3.50; *p* = 0.022). **Conclusions:** In neonates who have undergone surgical intervention for esophageal atresia, a risk factor for early postoperative complications is complications during delivery, while the risk factors for late postoperative complications are preoperative mechanical ventilation, postoperative sepsis, and belonging to relatively high- and high-risk groups according to the Spitz classification. Therefore, a multidisciplinary approach and continuous monitoring are essential to reduce morbidity and mortality, as well as to improve quality of life, in these patients.

## 1. Introduction

Esophageal atresia (EA) with tracheoesophageal fistula (TEF) is considered to be the most common congenital anomaly in the infant period that requires surgical intervention [1]. There are five anatomic variants of EA-TEF according to the Gross classification [1]. The potential complications associated with these conditions can increase the morbidity and mortality of affected patients [2]. The frequency of esophageal atresia is reported to be between 1 in 2500 and 1 in 4500 live births [3]. Previously, it has been reported that tracheoesophageal fistula is present in 70–90% of pediatric patients with esophageal atresia, while other systemic congenital anomalies have been reported in 55% of these children, particularly severe cardiac malformations, which might be associated with an unfavorable prognosis in these patients [4]. In patients with esophageal atresia, according to the Spitz classification, survival is associated with birth weight and the presence of major congenital heart defects [5].

With improvements in surgical interventions, the survival of patients with esophageal atresia has increased, with a survival rate greater than 90% reported [6]. However, this improvement is suggested to be multifactorial, including advances in neonatal intensive care and anesthesia, antibiotics, and ventilator and nutritional support, as well as surgical techniques and materials [7]. Despite improvements in survival, it is still pointed out that pediatric patients who have undergone surgical correction of esophageal atresia and tracheoesophageal fistulae experience significant morbidity into adulthood [6]. Additionally, in a study by Tuğba et al., postoperative early and late complications were reported in patients with esophageal atresia [8]. Moreover, in the literature, long-term digestive, respiratory, and neurological morbidity has been described, particularly after multi-stage esophageal repair in children with esophageal atresia [9].

In a study by Cho et al., it was stated that long-gap esophageal atresia was a significant risk factor for postoperative complications [10]. Furthermore, Rayyan et al. pointed out that prematurity in pediatric patients with esophageal atresia was a factor associated with a complicated clinical course during the early life period [11]. Additionally, it was reported that the predictive factors of complicated evolution in children with esophageal atresia during the first year of life were twin birth, birth weight below 2500 g, long-gap atresia, preoperative tracheal intubation, anastomotic leak, inability to be fed orally within the first month, and postoperative tracheal intubation for five or more days, while long-gap atresia and inability to be fed orally within the first month were shown to be associated with complicated evolution after the first year of life [12].

We hypothesized that certain factors might have an influence on onset of early and late postoperative complications in children with esophageal atresia. Therefore, the aim of our study was to evaluate the risk factors associated with early and late postoperative complications in neonatal patients with esophageal atresia.

## 2. Methods

### 2.1. Study Sample and Design

We performed a retrospective clinical observational study, with neonatal patients who were admitted between 1 January 2016 and 1 January 2025 to the University Children’s Hospital (UCH) in Belgrade, Serbia. The study sample comprised 109 neonatal patients aged between 0 and 27 days of life who were prenatally or at birth diagnosed with esophageal atresia. The data of the patients were collected from the hospital information system of the UCH, where a total of 113 patients were allocated during the defined study search period. The dropout was 4 patients (3.54%), since parents or legal guardians were not reached to provide informed consent for study participation. Prior to inclusion in this study, parents or legal guardians were informed about the study protocol and informed consent was obtained. This study was approved by the Institutional Ethics Review Board of the UCH (No. 16/89, date of approval 16 April 2025).

The UCH in Belgrade is a regional referral university hospital for diagnosis and treatment of neonates with congenital anomalies of the gastrointestinal tract, where most patients from Serbia, Montenegro, Bosnia and Herzegovina, and North Macedonia are referred for diagnosis and treatment.

All pediatric patients who were admitted to the UCH and diagnosed with esophageal atresia were surgically treated in the operating room. Those patients with short-gap were treated using primary repair, while long-gap was treated with staged approach surgery.

According to the revised Spitz classification, our patients were divided into 4 groups [13]:

1. Class 1 (low-risk group)—patients without major cardiac anomalies and birth weight > 2000 g.
2. Class 2 (moderate-risk group)—patients without major cardiac abnormalities and birth weight < 2000 g.
3. Class 3 (relatively high-risk group)—patients with major cardiac anomalies and birth weight > 2000 g.
4. Class 4 (high-risk group)—patients with major cardiac anomalies and birth weight < 2000 g.

Esophageal atresia was classified as follows [14]:

1. Type A (isolated EA).
2. Type B (EA with proximal TEF).
3. Type C (EA with distal TEF).
4. Type D (EA with proximal and distal fistulas).
5. Type E (H-type fistula).

Gestational age was classified according to delivery following the onset of the last menstrual period as follows [15]:

1. Preterm—up to 37 weeks.
2. Term—between 37–41 weeks.
3. Post-term—42 weeks and above.

The gap between two esophageal pouches was classified as follows [16]:

1. Short gap—gap length < 2 cm.
2. Long gap—gap length > 2 cm.

### 2.2. Study Variables

Further study variables were analyzed:

- Complications during delivery: present or absent. They included prelabor rupture of membranes (PROM), placenta previa, placental abruption, nuchal cord, polyhydramnios, RhD incompatibility, fetus breech position, chorioamnionitis.
- Perinatal asphyxia, which is defined according to the American Academy of Pediatrics (AAP) and American College of Obstetrics and Gynecology (ACOG) when all the following criteria are met: profound metabolic or mixed acidemia (pH < 7.00) in an umbilical arterial blood sample, Apgar score of 0–3 5 min after birth, neonatal encephalopathy (e.g., seizures, coma, hypotonia), and multiple organ involvement (kidneys, lungs, liver, heart, intestines) [17].
- Intracranial hemorrhage (ICH): present or absent. Cranial sonography that was performed by a Board-certified radiologist was used for diagnosis of intracranial hemorrhage.
- Respiratory distress: present or absent. It was defined as tachypnea (respiratory frequency (RF) > 60/min, nasal flaring, intercostal or subcostal retractions, audible grunting, and cyanosis) [18].
- Comorbidities of mother: present or absent. They included Hashimoto’s thyroiditis, hypertension, preeclampsia, gestational diabetes, diabetes mellitus, thrombophilia, anemia, arrhythmia, antiphospholipid syndrome, HELLP syndrome, and infection during pregnancy. Data of evaluated comorbidities were taken from the patient’s medical records.
- Applied medicamentous therapy during pregnancy—antiplatelet drugs: acetylsalicylic acid (ASA), nadroparin calcium, enoxaparin; antihypertensive drugs: methyldopa, verapamil, furosemide; antidiabetic drug: metformin; hormone drugs: progesterone, levothyroxine; antimicrobial drugs: ampicillin, gentamicin, trimethoprim/sulfamethoxazole, acyclovir; other: diazepam, ferrous supplement.
- Neonatal conditions: present or absent. They included cardiovascular: patent foramen ovale (PFO), patent ductus arteriosus (PDA), ventricular septal defect (VSD), atrial septal defect (ASD), mitral valve insufficiency (MR), persistent pulmonary hypertension in the neonate (PPHN), tetralogy of Fallot (TOF), total anomalous pulmonary venous connection (TAPVC), single umbilical artery (SUAS); respiratory: pneumonia, tracheomalacia, laryngotracheal cleft; urogenital: hypospadias, hydronephrosis, cryptorchidism, ectopic kidney, renal agenesis, horseshoe kidney, renal cyst, cloaca; musculoskeletal: clubfoot (talipes equinovarus), torticollis, scoliosis, polydactyly, syndactyly, finger aplasia, cleft lips and cleft palate, micrognathia; metabolic: icterus, hypothyroidism, glucose/galactose intolerance; neurological: spina bifida, convulsions, ventriculomegaly; syndromes: Down syndrome, Weaver syndrome, Townes–Brocks syndrome, Goldenhar syndrome. Diagnosis of neonatal conditions was made by a Board-certified pediatrician, pediatric surgeons, and physiatrist.
- Prenatal conditions: present or absent, including polyhydramnios and a small or absent stomach. These conditions were diagnosed using ultrasound performed by a certified radiologist; in cases with differential diagnostic dilemma, magnetic resonance imaging was performed.
- Preoperative mechanical ventilation (MV) (applied/not applied): The parameters for MV were Silverman score > 5; grades III and above of respiratory distress confirmed with chest radiography; failure of improvement with continuous positive airway pressure (cPAP) of 1 kPa at fraction of inspired oxygen (FiO_2_) 100%; periodical breathing with progressive decrease in base excess; progressive increase or irregular tachypnea with base excess decrease, partial fraction of oxygen (PaO_2_) > 6.6 kPa of FiO_2_ > 60%; hypoxemia from PaO_2_/FiO_2_ < 150 and score of acid/base balance grades III and above (PaO_2_ < 6.6 kPa, PaCO_2_ > 9.3 kPa, pH < 7.1) [19].
- Total parenteral nutrition (TPN): administered or not administered. The criteria for administration is expected not to be fed for more than seven days. The basic solution contained 20% to 25% dextrose and 3% to 5% crystalline amino acids from the commercially available kits/solutions [20,21].
- Postoperative sepsis: present or absent. All patients selected for this study were hospitalized for no fewer than 4 days [22]. The gold standard for the diagnosis of sepsis is a positive blood culture [23].

### 2.3. Postoperative Complications

- Early postoperative complications: those that appeared in the first month after surgery and included anastomotic dehiscence (“leak”), strictures, fistula, and short bowel syndrome [24].
- Late postoperative complications: those that appeared after the first month post surgery and included gastroesophageal reflux, ileus, tracheomalacia, respiratory infections, esophageal/intestinal peristaltic disorder, and chest deformities [24].

### 2.4. Statistical Analysis

Evaluated parameters are presented as whole numbers (N) and percentages (%). Statistical analyses between tested groups of categorical variables were performed using the chi-squared test and Fisher’s exact test. Univariate logistic regression analysis was performed for the tested variables, and those with significant findings were included in multivariate backward stepwise logistic regression models. To perform logistic regression analysis for groups according to the Spitz classification, two models were generated:

1. Model 1—included patients with Class 1 and Class 2 Spitz classification.
2. Model 2—included patients with Class 3 and Class 4 Spitz classification.

Statistical analysis was performed using IBM SPSS statistical software (SPSS for Windows, version 26.0, SPSS, Chicago, IL, USA). Statistical significance was set at *p* < 0.05.

## 3. Results

The frequencies of the tested variables in patients with esophageal atresia are presented in Table 1. Patients with complications during delivery (56.1%), neonatal conditions (85.2%), early (57.8%) and late (57.8%) complications, postoperative sepsis (54.6%), short gap (78.9%), and alternative roots of nutrition (92.6%) were more frequently presented. Those with Class 1 according to Spitz (63.9%) and EA type C (82.6%) were more frequently presented.

The distribution of the tested variables regarding presence of early postoperative complications in patients with esophageal atresia is presented in Table 2. Patients with early postoperative complications had significantly more frequent complications during delivery (*p* = 0.002), asphyxia (*p* = 0.038), applied medicamentous therapy (*p* = 0.035), and postoperative sepsis (*p* = 0.045).

The distribution of the tested variables regarding presence of late postoperative complications in patients with esophageal atresia is presented in Table 3. Patients with late postoperative complications had significantly more frequent complications during delivery (*p* = 0.025), respiratory distress (*p* = 0.043), preoperative mechanical ventilation (*p* = 0.014), and postoperative sepsis (*p* = 0.010). There was a significantly different distribution in frequencies of different classes of patients who were classified according to the Spitz classification between groups with and without late postoperative complications (*p* = 0.008).

Factors associated with early postoperative complications from univariate logistic regression analysis were complications during delivery (*p* = 0.003), perinatal asphyxia (*p* = 0.041), applied medicamentous therapy (*p* = 0.037), and postoperative sepsis (*p* = 0.046). Factors associated with late postoperative complications from univariate logistic regression analysis were complications during delivery (*p* = 0.026), respiratory distress (*p* = 0.045), preoperative mechanical ventilation (*p* = 0.016), postoperative sepsis (*p* = 0.011), and patients belonging to relatively high- and high-risk groups according to the Spitz classification (*p* = 0.033) (Table 4).

The multivariate backward stepwise logistic regression analysis regarding early and late postoperative complications in patients with esophageal atresia is presented in Table 4. The risk factor for early complications for patients with the upper type of atresia was complications during delivery (OR: 3.09; *p* = 0.007). The risk factors for late postoperative complications for patients with the upper type of atresia were preoperative mechanical ventilation (OR: 2.77; *p* = 0.041), postoperative sepsis (OR: 2.60; *p* = 0.028), and patients from relatively high- and high-risk groups according to the Spitz classification (OR: 3.50; *p* = 0.022).

## 4. Discussion

In our study on neonates with esophageal atresia, it was noticed that there were more males than females. Similar findings were reported previously, where in particular in the Global PaedSurg Research Collaboration, it was noticed that 56% of patients with esophageal atresia/tracheoesophageal fistula were males [25]. Additionally, more than four out of five patients from our study had type C of EA (82.6%), which is in line with previous reports [1]. Regarding postoperative complications, in our study, both early and late postoperative complications were reported in less than two thirds (57.8%, respectively) of neonates with esophageal atresia that underwent surgical intervention. A multicenter retrospective study of the Midwest Pediatric Surgery Consortium found 62% of infants with proximal esophageal atresia and distal tracheoesophageal fistula with postoperative complications [26].

Pediatric patients with early postoperative complications had more than 1.5 times more complications during delivery, just below 2 times the presence of asphyxia, below 2 times more applied medicamentous therapy, and below 1.5 times more postoperative sepsis compared to those without these types of complications. However, in multivariate backward stepwise logistic regression, we demonstrated that patients with early complications during delivery were 3.09 times more likely to have early postoperative complications.

Due to intensive multisystemic development during the neonatal period, certain affected organs can have influence treatment outcomes. Previously, it was reported that the most common early complication in neonates with esophageal atresia was pneumonia [27]. Furthermore, it was pointed out that perinatal asphyxia influences circulatory and respiratory responses as well as transitional circulatory changes, thus affecting cardio-respiratory adaptation [28]. Moreover, it was stressed that asphyxia can be considered as a major cause of morbidity and mortality in the neonatal period [29].

Pediatric patients with late postoperative complications in our study had more than 1.5 times more complications during delivery, around 1.5 times more respiratory distress, and more than 2 times more preoperative mechanical ventilation and postoperative sepsis compared to those without these types of complications. In multivariate backward stepwise logistic regression, we showed that patients with preoperative mechanical ventilation were 2.77 times more likely, and those with postoperative sepsis 2.60 times more likely, to have late postoperative complications.

Previously, it was noticed that for patients undergoing surgical intervention for esophageal atresia, postoperative sepsis is considered an important risk factor for mortality [27,30]. Furthermore, patients with sepsis are more susceptible to developing pneumonia, leading to respiratory dysfunction, thus increasing the need for mechanical ventilation [31]. Central venous catheter use and invasive medical devices are considered to be potential risk factors for sepsis [32]. This is important for neonatal patients undergoing surgical interventions. In a study by Dey et al., it was stressed that anastomotic leak may result from sepsis among other possible conditions including esophageal ischemia, poor suturing techniques, excess anastomotic tension, distal pouch extensive mobilization, and increased gap length [33]. Our results showed that postoperative sepsis was significantly more frequent in patients with early as well as late postoperative complications.

Furthermore, it was previously stated that pediatric patients with esophageal atresia and very low birth weight have a higher rate of complications [34]. In a study by van Helsdingen et al., the authors stated that low birth weight and major cardiac anomalies were associated with worse outcomes in patients with esophageal atresia [35]. Additionally, patients with esophageal atresia and extremely low birth weight were shown to have poor prognosis [36]. We have demonstrated that pediatric patients with esophageal atresia that belong to relatively high- and high-risk groups according to the Spitz classification are 3.50 times more likely to have late complications.

Our findings suggest that neonates following surgery for esophageal atresia should be continuously monitored in the postsurgical period in order to promptly recognize the potential complications and include patients with such complications in timely and adequate treatment.

Regarding medicamentous treatment, we have demonstrated that patients of mothers who were on applied medicamentous therapy had a significantly higher frequency of early postoperative complications, while such significance did not persist for late postoperative complications. Neonates born to mothers taking regular pharmacological treatment for chronic conditions during pregnancy, including but not limited to antihypertensive therapy, insulin or oral hypoglycemics for diabetes mellitus, and thyroid hormone replacement, could have a higher incidence of early postoperative complications [37]. This phenomenon might result from a complex interplay between maternal pathology, drug transfers through the placenta, and consequent organ immaturity [37]. Early postoperative complications in neonates whose mothers received medicamentous therapy during pregnancy are more common, as maternal medications may affect neonatal vulnerability. This is particularly relevant in cases of esophageal atresia, where more than 80% of neonates underwent surgical intervention within the first three days of life, while the pharmacokinetic effects of the administered drugs may still be present and exert influence [38].

## 5. Limitations

This study was performed in a single center and included a small number of patients. Thus, further studies should be conducted on a larger number of participants to increase the sensitivity of the obtained findings.

## 6. Conclusions

In neonates who underwent surgical intervention for esophageal atresia, risk factors for early postoperative complications are complications during delivery, while risk factors for late postoperative complications are preoperative mechanical ventilation, postoperative sepsis, and patients belonging to relatively high- and high-risk groups according to the Spitz classification.

The increased risk of complications in neonates with esophageal atresia after surgery highlights the necessity for a multidisciplinary approach in prenatal and postnatal diagnostics and treatment along with continuous monitoring to reduce morbidity and mortality and improve overall quality of life.

## Figures and Tables

**Table 1:** Frequencies of tested variables in patients with esophageal atresia.

Variables		N (%)	Response Rate, %
Gender	Male	66 (60.6)	100%
	Female	43 (39.4)	
Complications during delivery	None	47 (43.9)	98.17%
	Yes	60 (56.1)	
Perinatal asphyxia	No	76 (69.7)	100%
	Yes	33 (30.3)	
Intracranial hemorrhage	No	91 (84.3)	99.08%
	Yes	17 (15.7)	
Respiratory distress	No	54 (49.5)	100%
	Yes	55 (50.5)	
Comorbidities of mother	No	65 (59.6)	100%
	Yes	44 (40.4)	
Applied medicamentous therapy	No	70 (64.8)	99.08%
	Yes	38 (35.2)	
Neonatal conditions	No	16 (14.8)	99.08%
	Yes	92 (85.2)	
Prenatal diagnostics	No	72 (66.1)	100%
	Yes	37 (33.9)	
Preoperative mechanical ventilation	No	73 (67.6)	99.08%
	Yes	35 (32.4)	
Total parenteral nutrition	No	8 (7.4)	99.08%
	Yes	100 (92.6)	
Early postoperative complications	No	46 (42.2)	100%
	Yes	63 (57.8)	
Late postoperative complications	No	46 (42.2)	100%
	Yes	63 (57.8)	
Postoperative sepsis	No	49 (45.4)	99.08%
	Yes	59 (54.6)	
Gestational age	Preterm	52 (47.7)	100%
	Term	57 (52.3)	
	Post-term	0 (0)	
Gap	Short	86 (78.9)	100%
	Long	23 (21.1)	
Spitz classification	Class 1	69 (63.9)	99.08%
	Class 2	11 (10.2)	
	Class 3	14 (13.0)	
	Class 4	14 (13.0)	
EA type	A	14 (12.8)	100%
	B	0 (0)	
	C	90 (82.6)	
	D	5 (4.6)	
	E	0 (0)	

EA—esophageal atresia.

**Table 2:** Distribution of tested variables regarding presence of early postoperative complications in patients with esophageal atresia.

Variables		Early Postoperative Complications		*p*
		No	Yes	
Gender	Male	28 (60.9)	38 (60.3)	0.954 *
	Female	18 (39.1)	25 (39.7)	
Complications during delivery	None	27 (61.4)	20 (31.7)	0.002 *
	Yes	17 (38.6)	43 (68.3)	
Perinatal asphyxia	No	37 (80.4)	39 (61.9)	0.038 *
	Yes	9 (19.6)	24 (38.1)	
Intracranial hemorrhage	No	38 (82.6)	53 (85.5)	0.685 *
	Yes	8 (17.4)	9 (14.5)	
Respiratory distress	No	25 (54.3)	29 (46.0)	0.391 *
	Yes	21 (45.7)	34 (54.0)	
Comorbidities of mother	No	30 (65.2)	35 (55.6)	0.310 *
	Yes	16 (34.8)	28 (44.4)	
Applied medicamentous therapy	No	35 (76.1)	35 (56.5)	0.035 *
	Yes	11 (23.9)	27 (43.5)	
Neonatal conditions	No	8 (17.4)	8 (12.9)	0.516 *
	Yes	38 (82.6)	54 (87.1)	
Prenatal diagnostics	No	32 (69.6)	40 (63.5)	0.508 *
	Yes	14 (30.4)	23 (36.5)	
Preoperative mechanical ventilation	No	34 (73.9)	39 (62.9)	0.227 *
	Yes	12 (26.1)	23 (37.1)	
Total parenteral nutrition	No	4 (8.7)	4 (6.5)	0.721 **
	Yes	42 (91.3)	58 (93.5)	
Postoperative sepsis	No	26 (56.5)	23 (37.1)	0.045 *
	Yes	20 (43.5)	39 (62.9)	
Gestational age	Preterm	21 (45.7)	31 (49.2)	0.714 *
	Term	25 (54.3)	32 (50.8)	
Gap	Short	38 (82.6)	48 (76.2)	0.417 *
	Long	8 (17.4)	15 (23.8)	
Spitz classification	Class 1	30 (65.2)	39 (62.9)	0.601 **
	Class 2	6 (13.0)	5 (8.1)	
	Class 3	6 (13.0)	8 (12.9)	
	Class 4	4 (8.7)	10 (16.1)	
EA type	A	3 (6.5)	11 (17.5)	0.282 **
	C	41 (89.1)	49 (77.8)	
	D	2 (4.3)	3 (4.8)	

*—chi-squared test; **—Fisher’s exact test; EA—esophageal atresia.

**Table 3:** Distribution of tested variables regarding presence of late postoperative complications in patients with esophageal atresia.

Variables		Late Postoperative Complications		*p*
		No	Yes	
Gender	Male	30 (65.2)	36 (57.1)	0.394 *
	Female	16 (34.8)	27 (42.9)	
Complications during delivery	None	25 (56.8)	22 (34.9)	0.025 *
	Yes	19 (43.2)	41 (65.1)	
Perinatal asphyxia	No	36 (78.3)	40 (63.5)	0.097 *
	Yes	10 (21.7)	23 (36.5)	
Intracranial hemorrhage	No	40 (88.9)	51 (81.0)	0.264 *
	Yes	5 (11.1)	12 (19.0)	
Respiratory distress	No	28 (60.9)	26 (41.3)	0.043 *
	Yes	18 (39.1)	37 (58.7)	
Comorbidities of mother	No	31 (67.4)	34 (54.0)	0.158 *
	Yes	15 (32.6)	29 (46.0)	
Applied medicamentous therapy	No	33 (73.3)	37 (58.7)	0.117 *
	Yes	12 (26.7)	26 (41.3)	
Neonatal conditions	No	9 (19.6)	7 (11.3)	0.231 *
	Yes	37 (80.4)	55 (88.7)	
Prenatal diagnostics	No	30 (65.2)	42 (66.7)	0.875 *
	Yes	16 (34.8)	21 (33.3)	
Preoperative mechanical ventilation	No	37 (80.4)	36 (58.1)	0.014 *
	Yes	9 (19.6)	26 (41.9)	
Total parenteral nutrition	No	6 (13.0)	2 (3.2)	0.070 **
	Yes	40 (87.0)	60 (96.8)	
Postoperative sepsis	No	27 (60.0)	22 (34.9)	0.010 *
	Yes	18 (40.0)	41 (65.1)	
Gestational age	Preterm	22 (47.8)	30 (47.6)	0.983 *
	Term	24 (52.2)	33 (52.4)	
Gap	Short	34 (73.9)	52 (82.5)	0.276 *
	Long	12 (26.1)	11 (17.5)	
Spitz classification	Class 1	30 (65.2)	39 (62.9)	0.008 **
	Class 2	9 (19.6)	2 (3.2)	
	Class 3	2 (4.3)	12 (19.4)	
	Class 4	5 (10.9)	9 (14.5)	
EA type	A	4 (8.7)	10 (15.9)	0.574 **
	C	40 (87.0)	50 (79.4)	
	D	2 (4.3)	3 (4.8)	

*—chi-squared test; **—Fisher’s exact test; EA—esophageal atresia.

**Table 4:** Logistic regression analysis regarding early and late postoperative complications in patients with esophageal atresia.

Variables	Logistic Regression			
	Early Postoperative Complication		Late Postoperative Complications	
	OR (95% CI)	*p*	OR (95% CI)	*p*
Univariate logistic regression				
Gender	1.02 (0.70–2.23)	0.954	1.41 (0.64–3.09)	0.395
Complications during delivery	3.42 (1.53–7.65)	0.003	2.45 (1.11–5.40)	0.026
Perinatal asphyxia	2.53 (1.04–6.15)	0.041	2.07 (0.87–4.93)	0.101
Intracranial hemorrhage	0.81 (0.29–2.28)	0.685	1.88 (0.61–5.78)	0.269
Respiratory distress	1.40 (0.65–2.99)	0.392	2.21 (1.02–4.81)	0.045
Comorbidities of mother	1.50 (0.69–3.29)	0.311	0.57 (0.26–1.25)	0.160
Applied medicamentous therapy	2.46 (1.06–5.70)	0.037	1.93 (0.84–4.43)	0.120
Neonatal conditions	1.42 (0.49–4.12)	0.518	1.91 (0.65–5.58)	0.236
Prenatal diagnostics	1.31 (0.58–2.96)	0.509	0.94 (0.42–2.09)	0.875
Preoperative mechanical ventilation	1.67 (0.72–3.85)	0.229	2.97 (1.22–7.20)	0.016
Total parenteral nutrition	1.38 (0.33–5.84)	0.661	4.50 (0.87–23.42)	0.074
Postoperative sepsis	2.20 (1.01–4.80)	0.046	2.80 (1.27–6.16)	0.011
Gestational age	0.87 (0.41–1.86)	0.714	1.01 (0.47–2.16)	0.983
Gap	1.48 (0.57–3.87)	0.419	0.60 (0.24–1.51)	0.278
Spitz class	0.90 (0.38–2.18)	0.822	2.85 (1.09–7.46)	0.033
Multivariate backward stepwise logistic regression				
Complications during delivery	3.09 (1.36–7.02)	0.007	-	-
Applied medicamentous therapy	2.19 (0.91–5.28)	0.080	-	-
Preoperative mechanical ventilation	-	-	2.77 (1.04–7.36)	0.041
Postoperative sepsis	-	-	2.60 (1.11–6.11)	0.028
Spitz class	-	-	3.50 (1.20–10.21)	0.022

## Data Availability

Original data are available on request from the corresponding author.
